# Cellular defense mechanisms against asbestos fibers

**DOI:** 10.3389/fpubh.2025.1566473

**Published:** 2025-05-14

**Authors:** Christy A. Barlow, Brooke T. Mossman

**Affiliations:** ^1^Aetia Scientific Collaborative, Boulder, CO, United States; ^2^Department of Pathology and Laboratory Medicine, Larner College of Medicine, University of Vermont, Burlington, VT, United States

**Keywords:** asbestos, elongated mineral fibers (EMPs), mesothelioma, lung cancer, threshold

## Abstract

Although inhalation of sufficient doses and dimensions of airborne asbestos dusts in an occupational setting can produce cancer in the lungs, pleura and peritoneum, tumors occur in <5–10% of exposed individuals, even among persons with considerable historical exposures. In this perspective, we review cell defense mechanisms that are involved in protective and adaptive responses to asbestos exposure. These adaptive responses are orchestrated through a multifaceted cellular program involving the concerted action of diverse stress response pathways, including antioxidant responses, DNA repair mechanisms, molecular mechanisms for intracellular signaling leading to proliferation, apoptosis, and inflammation, and cell cycle regulation. These cell defenses suggest that humans can adjust to moderate levels of stress or change without experiencing negative effects, implying the existence of a threshold dose. Likewise, reported no-observed adverse-effect levels (NOAELs) for various asbestos fiber types and asbestos-related cancers in experimental and epidemiological data further support the existence of a threshold dose and are discussed here.

## 1 Introduction

Asbestos is a generic term for a family of naturally occurring hydrated silicate minerals (1, 2). It encompasses a group of six chemically and physically diverse types of asbestiform minerals that are characterized according to morphology as serpentine (chrysotile) or amphibole (crocidolite, amosite, tremolite, anthophyllite, and actinolite). Chrysotile, the sole member of the serpentine group, is a hydrated magnesium silicate that is represented by the chemical formula Mg_3_[Si_2_O_5_](OH)_4_, whereas the two most commercially important amphiboles, crocidolite and amosite, have a high iron content and are represented as $\text{N}{\text{a}}_{2}\text{F}{\text{e}}_{2}^{3+}\text{F}{\text{e}}_{3}^{2+}[\text{S}{\text{i}}_{8}{\text{O}}_{22}]{\left(\text{OH}\right)}_{2}$ and $\text{F}{\text{e}}_{7}^{2+}[\text{S}{\text{i}}_{8}{\text{O}}_{22}]{\left(\text{OH}\right)}_{2}$, respectively (3). It should be acknowledged that increased risks of both lung cancers and malignant mesotheliomas (MMs) have been observed in workers exposed to asbestos fibers occupationally, but the carcinogenic potency of commercially used fibers (crocidolite > amosite > chrysotile) is vastly different, especially in the development of MMs (4–10).

Asbestos-related disease conforms to standard toxicological principles for human disease and exhibits a non-linear dose-response relationship (8). Scientific data increasingly suggest that promoters and other non-genotoxic agents will have a non-linear dose-response relationship at low doses (11, 12). Non-linear relationships and the assumption that no increase in risk occurs at low doses of agents below a certain level have been endorsed for ionizing radiation and chemicals in general, providing evidence for thresholds for both DNA interacting and epigenetic chemical carcinogens (13–19).

Humans have evolved multiple defense mechanisms against the external environment to maintain homeostasis within their internal environment. In response to asbestos and other carcinogens, defensive and adaptive cellular responses exist to prevent cellular injury and permit normal cell survival. Adaptive responses include metabolism and elimination of foreign agents, as well as damage repair that can occur at both the cellular and molecular level. Formation of cancers is thought to involve a loss in multiple adaptive cellular responses (20). In this perspective, we review cell defense mechanisms that are involved in protective and adaptive responses to asbestos fiber exposures in lung epithelial and mesothelial cells, target cells of asbestos-induced lung carcinomas and MMs. Then, we review experimental and epidemiological evidence suggesting thresholds for exposures to asbestos fibers. Knowledge of these defense responses are important in the regulation of asbestos exposures and the design of preventive and therapeutic approaches to both lung cancers and MMs.

## 2 Cell defense/adaptive responses

Researchers have used *in vitro* studies on isolated cells and organ cultures to address key events in carcinogenesis by asbestos (21). These *in vitro* models allow for the measurement of acute responses, including those important in defense and adaptation, after exposure to known concentrations of fibers over a range of doses. In this perspective, *in vitro* data, have been correlated, whenever possible, to events occurring after inhalation, the natural route of access to the body, of various asbestos fiber types in rodents and human lungs (22). There are several limitations in many of the studies described below including a general lack of dose-response experiments and the lack of inclusion of non-disease causing particles or fibers (negative controls) to determine the specificity of asbestos-induced responses. Additionally, most animal studies evaluated effects attributed to asbestos on an equal mass or weight basis even though the number, surface area and reactivity of fibers at equal weight concentrations may be vastly different, thereby rendering comparisons between different experiments and human exposure difficult.

Adaptive cellular responses are of specific interest in this perspective and include multiple DNA repair mechanisms; a battery of antioxidant responses; proteins that monitor cellular proliferation, growth, and adhesion; and cell cycle check point mechanisms that pause cell proliferation to allow for DNA repair.

### 2.1 DNA repair mechanisms

Since a consequence of asbestos-induced oxidative damage may include DNA single strand breaks (SSB) and oxidant-induced 8-hydroxydeoxyguanosine (8-OHdG) DNA adducts (23, 24), it is important to discuss the human physiological response to such insult. In mammalian cells, four major DNA damage repair mechanisms are known to be responsible for repairing different DNA lesions: base excision repair (BER), nucleotide excision repair, mismatch repair, and recombinational system repair (25). Polymorphisms in genes encoding DNA repair proteins, such as XRCC1 and BRCA1, have also been suggested to be associated with the risk of MM (26, 27). Moreover, germline mutations in DNA repair genes may induce MMs (28) or predispose asbestos-exposed individuals to MMs (29, 30).

Different types of asbestos have been shown to produce 8-OHdG DNA adducts, DNA damage, and/or chromosomal aberrations in mesothelial cells *in vitro* and *in vivo* (23, 24). Adduct formation, oxidative damage, alkylation, and deamination can all create DNA base damage that is repaired by the same base excision repair mechanism. This pathway is also responsible for repairing DNA SSBs generated by reactive oxygen species (ROS) (31). DNA glycosylases recognize and remove the damaged base leaving a site that that is absent of a purine and pyrimidine (AP site) (32). The AP site is repaired by an AP-endonuclease and ligase which initially creates a gap in the DNA strand that is eventually filled in by DNA polymerase and DNA ligase (33). AP-endonuclease co-localizing with mitochondria is increased in mesothelial cells after exposure to crocidolite asbestos, suggesting that repair mechanisms exist (34). Moreover, mitochondrial 8-oxoguanine DNA glycosylase, a mtDNA BER enzyme, reduces mtDNA damage and apoptosis by amosite in alveolar epithelial cells and mitigates lung fibrosis (35).

Arguably, DSB are the most significant form of DNA damage and could lead to cell lethality and transformation if left unrepaired (36). As few as one DSB is sufficient to kill a cell if it inactivates an essential gene or induces apoptosis. Mammalian cells have two somewhat redundant mechanisms for repairing DSBs: homology-directed repair and non-homologous end-joining repair (33). The pathway that the cell uses to repair the DSB depends on the phase of cell cycle when the error is detected and the type of DNA lesion. The non-homologous end-joining mechanism is used if the cell is in the G1 phase of cell cycle. If the cells are in S or G2 phase of the cell cycle, then the cell repairs the DSB by the homology-directed repair mechanism (37). Marczynski et al. reported that there are higher incidences of DSBs in white blood cells of occupationally exposed asbestos workers as compared with the non-exposed control population (38).

The upregulation of DNA repair genes after exposure to asbestos is a strong indication of an adaptive response, meaning that a cell is actively increasing its ability to repair DNA damage in response to a stressful environment, allowing it to better tolerate and survive the damaging conditions. This upregulation is often part of a broader cellular response to stress, where the cell activates various mechanisms to mitigate damage and maintain homeostasis.

### 2.2 Antioxidant responses

Despite decades of research on the health effects of asbestos, the underlying mechanisms leading to asbestos-induced pulmonary toxicity and cancers are not completely understood; however, much research has focused on the importance of generation of ROS and reactive nitrogen species (RNS). Multiple mechanisms of generation of oxidant species occur in lung epithelial cells, mesothelial cells, and phagocytes, such as alveolar and pleural macrophages, after exposure to amphibole asbestos and other fibers (39, 40). For example, the high iron content and iron availability of amosite and crocidolite asbestos generates a Haber-Weiss reaction producing superoxide and hydrogen peroxide that may function extracellularly or within cells after fiber uptake. Whereas, cells of a variety of types can internalize fibers less than their cell diameters, longer fibers cause frustrated phagocytosis which stimulates NADPH (nicotinamide adenine dinucleotide phosphate) oxidase in cell membranes, a potent enzyme in production of ROS. Once internalized into an acidic phagolysosome, shorter fibers undergo changes in composition and structure (41) and exist in perinuclear vesicles or free in the cytoplasm (42).

Mitochondria are both a source and target of ROS generated by crocidolite asbestos in mesothelial and pulmonary epithelial cells. Oxidant elaboration results in damage to mitochondrial DNA (mtDNA) and dose-related alterations in mitochondrial gene expression (43–45). At high toxic fiber concentrations, apoptosis occurs. The importance of oxidative stress in asbestos-induced toxicity and cell death has been discussed previously (39, 40).

In addition to ROS- and RNS-induced protein modifications *in vitro* and *in vivo*, DNA and lipids undergo structural changes that are linked to damage of macromolecules and cell death at high concentrations of asbestos and initiation of multiple signaling pathways linked to carcinogenic events at low concentrations. Signatures of oxidative RNA and DNA damage have been observed for as long as 72 h in human mesothelial cells at growth inhibitory concentrations of crocidolite (34, 46), in isolated rat pleural mesothelial cells at both toxic and non-toxic concentrations of crocidolite (34), and in rats after intraperitoneal injection of crocidolite (47).

Since amosite and crocidolite asbestos are high iron-containing and the most potent in the induction of human mesotheliomas, most studies have focused on their mechanisms of oxidant stress and defense (40, 48, 49). Surface active sites reducing O_2_ and catalyzing the decomposition of H_2_O_2_ to reactive radicals, such as ^•^OH, or lipid radicals are largely related to the presence and ionic state of iron. Moreover, pre-addition or co-administration of iron chelators or catalase inhibits cell injury, inflammation, and asbestosis *in vivo* (50, 51), supporting a direct role of iron in lung disease.

One well-documented protective response to longer (generally > 10 or 20 microns) amphibole fibers is the formation of asbestos (ferruginous) bodies in the lung that are comprised of predominately ferric iron with minor amounts of protein and mucopolysaccharides. The sequestration of fibers in a non-reactive core is an important defense mechanism as the valence sites of ferric moieties are not available for electron exchange and generation of ROS. In addition, asbestos bodies, in comparison to native fibers, show both diminished formation of ROS and reduced toxicity (49).

Upregulation and increased expression of a cadre of antioxidant enzymes have also been demonstrated *in vitro* and *in vivo* after exposures to asbestos fibers and inflammatory particles (52). Conventional antioxidant enzymes include copper–zinc containing superoxide dismutase (CuZnSOD or SOD1), manganese-containing superoxide dismutase (MnSOD or SOD2), catalase, and glutathione peroxidase (GPX) and may act alone or in combination to prevent asbestos-associated injury (51, 53). Other proteins, including heme oxygenase-1 (HO-1), GRP78, and heat shock proteins (HSP70), are linked to prevention of oxidant stress and increase after exposures of lung epithelial and mesothelial cells to crocidolite fibers *in vitro* (54, 55).

Supplementation of antioxidant enzymes also inhibits asbestos-induced toxicity and signatures of carcinogenesis. For example, in tracheobronchial epithelial cells, minimally toxic concentrations of crocidolite or chrysotile asbestos caused protracted increases in total SOD levels, and toxicity was ameliorated by addition of SOD (56). In rat lungs and human bronchi, CuZnSOD was prominent in macrophages and bronchiolar epithelial cells, suggesting protective mechanisms in both target cells of lung cancers and effector cells of the immune system. Administration of antioxidant enzymes inhibited crocidolite-induced cell injury, inflammation, and fibrotic changes (51). In comparison to other antioxidant enzymes, mitochondrial MnSOD was most strikingly elevated, and linked to injury and inflammation of other fibrogenic minerals such as silica (57–59). Transfection of MnSOD into tracheal epithelial cells exposed to asbestos inhibited asbestos-induced toxicity (60). Individuals with a GSTM1 null allele and Ala/Ala genotypes of codon 16 within the MNSOD gene exhibit increased risk of MM (61).

Cell glutathione and thiol levels are critical defense mechanisms after oxidant stress by asbestos (62, 63). Depletion of total cell glutathione pools occurs after addition of crocidolite to mesothelial cells and is accompanied by increased levels of *c-fos* and *c-jun* early response prot(o)oncogenes (64). Addition of N-acetyl-cysteine to boost thiol levels decreased asbestos-mediated gene expression in a dose-dependent fashion (64).

The thioredoxin system is composed of NADPH, cytosolic and mitochondrial thioredoxins, and thioredoxin reductases. Crocidolite asbestos oxidized the pool of the antioxidant, Thioredoxin-1 (TRX1), in human mesothelial cells via production of ROS (65). Modulation of thioredoxins and thioredoxin interacting protein (TXNIP) showed that TRX1 overexpression or knockdown of TXNIP attenuated NLRP3 inflammasome activation, reinforcing the role of inflammasome activation by oxidants and subsequent generation of proteins, IL-1B and HMGB1, linked to mesothelioma development (66, 67).

Nuclear Factor Erythroid 2-Related Factor 2 (Nrf2) is a transcription factor that plays a key role in controlling the inducible expression of enzymes linked to the synthesis of glutathione and other antioxidants. As such, it is important in the control of inflammation (68). When exposed to ROS, Nrf2 migrates from the cytoplasm to the nucleus where it leads to the upregulation of expression of several antioxidant and detoxification genes (e.g., GSTs, SODs, and HO-1), the downregulation of NF-κB, and reduction in proinflammatory cytokines (e.g., IL-6 and IL-1β). This is an adaptive mechanism that enhances resiliency in response to subthreshold doses of toxins.

In summary, cells of the immune system and target cells of asbestos-associated diseases exhibit oxidative stress that can be counteracted by antioxidant responses at low concentrations of asbestos fibers that are non-toxic but are overwhelmed at high fiber concentrations that may be carcinogenic or cause cell death.

### 2.3 Molecular mechanisms of proliferation and inflammation

**Figure 1:**
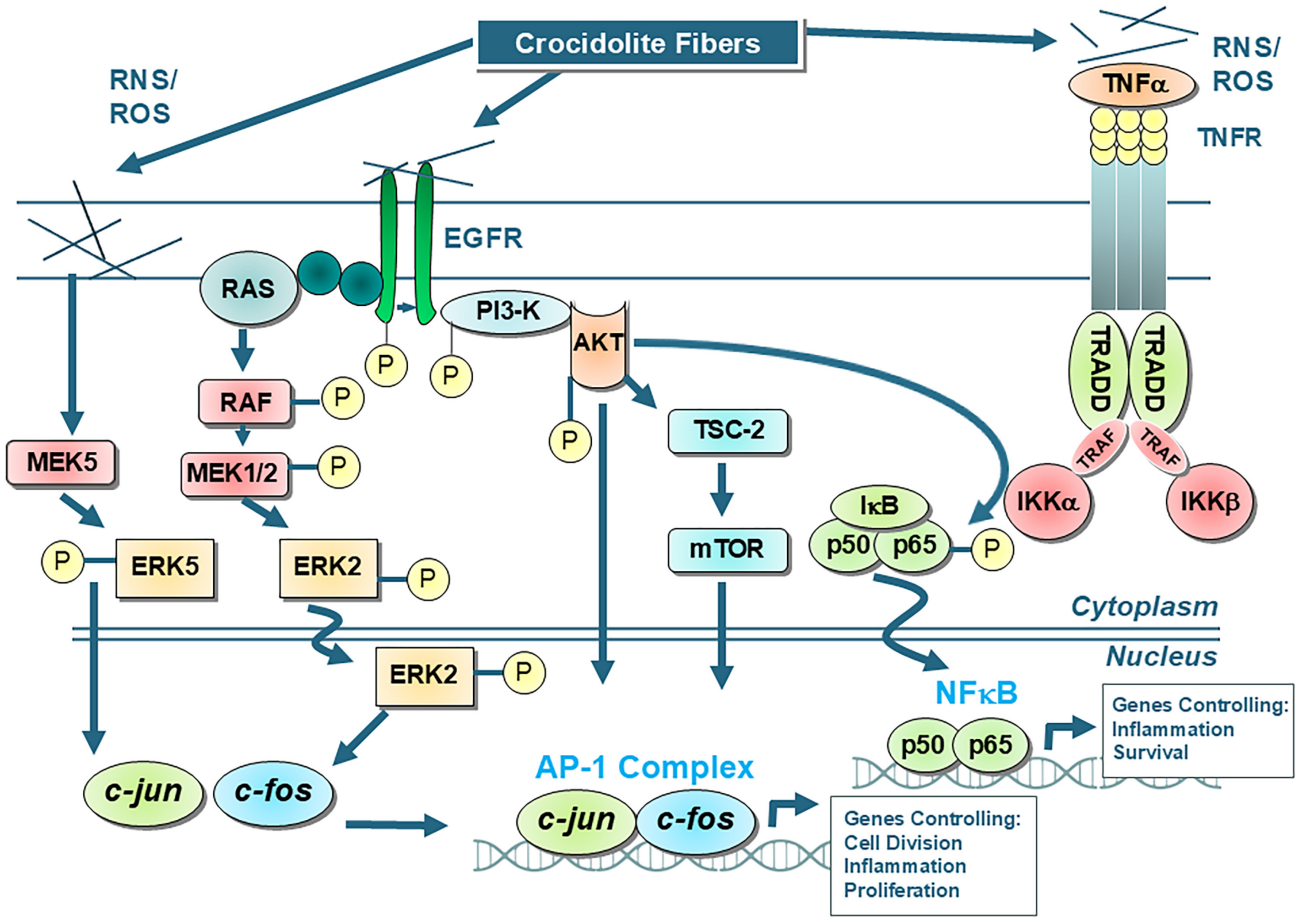
Shows the main AP-1 and NF-kB cell signaling pathways that occur in tracheobronchial/lung epithelial and mesothelial cells after exposures to asbestos fibers, such as amosite and crocidolite asbestos, that initially interact with the external cell membrane. Interactions of fibers or elaboration of oxidants then result in activation of protein cascades that cause alterations in gene expression. It is unlikely that asbestos and other elongated mineral fibers at low concentrations induce cancerous changes by interacting directly with the DNA of cells. For example, the mutagenic changes reported using chrysotile asbestos in a human/hybrid cell model are observed at high concentrations causing large scale deletions incompatible with cell survival (40, 140). Moreover, crocidolite and erionite induce polyploidy and clastogenic effects in cells, but not mutations (183). In contrast to chrysotile asbestos which dissolves over time in rodent and human lungs (139, 184), durable amosite and crocidolite fibers produce many of the hallmarks of cancers, most notably genomic instability, sustained cell proliferation and chronic inflammation (13, 69, 185). These and other characteristics of cancer observed in asbestos-associated cell outcomes are attributed to long, iron-rich amphibole fibers, altered cell signaling events, and production of reactive oxygen and nitrogen species (ROS, RNS) (69).

Both Activator Protein-1 (AP-1) and NF-κB are redox-acivated transcription factors that play crucial roles in regulating cellular processes such as proliferation and inflammation by controlling gene expression. These events are hallmarks of the cancer process (69). Generally, frustrated phagocytosis of asbestos fibers triggers the production of ROS within cells, which in turn activates cellular signaling pathways leading to the phosphorylation and nuclear translocation of AP-1 and NF-κB subunits (Figure 1). These events can lead to altered gene expression affecting cellular processes linked to proliferation, altered cell function (metaplasia), cell death, and inflammation. In general, a relatively low concentration of chrysotile or amphibole asbestos exposure (<1 μm/cm^2^) results in proliferative signaling in mesothelial cells, while higher asbestos concentrations result in apoptosis and block proliferation (70–74). Proliferation of mesothelial cells by crocidolite asbestos, the phorbol ester tumor promoter, TPA (12-O-tetradecanoylphorbol-13-acetate), and TNFα (Tumor Necrosis Factor alpha) suggest multiple cell pathways leading to tumor promotion in MMs (75). In contrast, apoptosis is an important protective mechanism by which DNA damaged cells are eliminated without initiating an inflammatory response (76). Likewise, acute inflammation is considered a vital defense mechanism as it is the immune system's response to harmful stimuli, like infections or injury, by ultimately restoring tissue homeostasis.

#### 2.3.1 TP53

TP53 (p53) acts as a tumor suppressor protein that generally functions to suppress inflammatory responses by negatively regulating NF-κB signaling (77). NF-κB can suppress p53 levels by upregulating the expression of MDM2, a target gene of NF-kB.

Studies have shown increased p53 protein expression occurs in lung epithelial and mesothelial cells and in rodents exposed to asbestos (78–80). In chrysotile-exposed mice and rats, p53 increases are observed prior to reversible increases in lung epithelial cell proliferation (81, 82). p53 primarily functions as a transcription factor that regulates a wide spectrum of genes including apoptosis, cell cycle arrest, senescence, autophagy, DNA repair, and angiogenesis (83). Broadly, p53 is a tumor suppressor that limits cellular proliferation by inducing cell cycle arrest and apoptosis in response to cellular stresses, such as DNA damage.

Beyond its traditional role as a transcription factor, recent research has shown that p53 can also directly translocate to the mitochondria under stress conditions, where it can interact with mitochondrial proteins to disrupt their function and initiate apoptosis (84). Amphibole asbestos exposure causes mitochondrial dysfunction, which leads to cell oxidative stress and apoptosis through the p53-regulated mitochondrial pathway (85). Moreover, spontaneous p53 mutations in murine mesothelial cells increase their sensitivity to crocidolite asbestos and ionizing radiation (86).

Loss of function mutations in p53 have been associated with the development of MMs in humans and rodents (87, 88). A select group of MM patients demonstrate mutations in the p53 gene (89).

#### 2.3.2 Activator protein-1 (AP-1)

Members of the Mitogen-Activated Protein Kinase (MAPK) family are critical to most aspects of AP-1 regulation. *In vitro* experiments have shown that the MAPK cascade is involved in both apoptotic and proliferative responses to abestos. Although the mechanism of action remains elusive, it has been shown that after interaction with cells, asbestos fibers trigger a series of multiple protein phosphorylation events occurring after asbestos-associated oxidant generation. After dimerization and rapid dissociation or phosphorylation of the the epidermal growth factor receptor (EGFR) (90), crocidolite asbestos fibers trigger many signaling cascades, including the AKT pathway and MAPK (73, 91–96). MAPKs consist of three major families: extracellular signal-regulated kinases (ERKs), c-Jun-NH2-terminal kinases (JNKs), and p38 kinases. ERKs (ERK1, 2, and 5) are effectors of the Ras proto-oncoprotein and are activated in response to mitogenic stimuli whereas JNKs and p38s are stress activated protein kinases that are activated preferentially by environmental stresses and inflammatory cytokines of the TNF family (97, 98). Asbestos has been shown to stimulate predominately ERKs, but also p38, and JNK pathways in alveolar type II epithelial cells and mesothelial cells (74, 99, 100). These oxidant regulated signaling pathways regulate gene expression of early response proto-oncogenes (*fos/jun* family) in mesothelial and lung epithelial cells (101, 102). Their proteins can dimerize, forming the transcription factor, AP-1 that interacts with DNA (103). These events may be linked to increases in early-response genes which govern cellular responses, such as proliferation and apoptosis (71). AP-1 activation also leads to increased production of pro-inflammatory cytokines (e.g., TNFα and IL-6) in certain cell types (104).

Since MAPKs are regulated by reversible phosphorylation on threonine and tyrosine residues, deactivation of MAPKs can also occur via dephosphorylation at these residues. This is the role and function of the MAPK phosphatases (MKPs) (105). MKP-1 specifically targets the phosphorylated forms of p38 and JNK, removing the phosphate group and rendering them inactive. By controlling p38 and JNK signaling, MKP-1 plays a crucial role in cell survival by regulating p38 and JNK in response to cellular stress. MKP-1 is upregulated in response to exposure to asbestos in human mesothelial cells (106).

#### 2.3.3 Nuclear factor-kappa B (NF-κB)

NF-κB is a redox-sensitive transcription factor and can be a downstream target of MAPK signaling pathways. Studies have shown that asbestos fibers activate NF-κB in tracheal epithelial and mesothelial cells *in vitro* and in rat lungs *in vivo* after asbestos inhalation (107–110). NF-κB regulates multiple aspects of innate and adaptive immune functions and serves as a pivotal mediator of inflammatory responses (111–113). NF-κB is comprised of protein dimers, including the transcription-activating heterodimer consisting of p50 and p65 (RelA) subunits. It is a ubiquitous redox-regulated transcription factor that is retained in the cytoplasm by forming an inactive complex with its cytosolic repressor, IkB. Oxidative and pro-inflammatory stimuli activate NF-κB through phosphorylation-dependent proteasomal degradation of IkBα, ultimately allowing NF-κB to move into the nucleus where it can influence gene expression. NF-κB induces the expression of various pro-inflammatory genes, including those encoding cytokines and chemokines (e.g., TNFα, IL-6, COX-2). It also participates in inflammasome regulation (114). In addition, NF-κB plays a critical role in regulating the survival, activation and differentiation of innate immune cells and inflammatory T cells.

#### 2.3.4 NLRP3 inflammasome

The NLRP3 inflammasome is a multiprotein complex that plays a pivotal role in regulating the innate immune system and inflammatory responses by interacting with various cell death pathways like apoptosis, pyroptosis, and necroptosis (115). By modulating these pathways, inflammation can be mitigated, and tissue repair and regeneration can be promoted.

The activation of the NLRP3 inflammasome is a two-step process, requiring a priming signal that upregulates NLRP3 expression and a subsequent activation signal that triggers the assembly of the inflammasome complex, leading to the maturation and release of pro-inflammatory cytokines like IL-1β and IL-18 (111, 115). Multiple studies have shown that crocidolite asbestos fibers prime and activate the NLRP3 inflammasome in human mesothelial and macrophage cells *in vitro* and in mice using inhalation models (66, 116, 117). Both AP-1 and NF-κB have been shown to play a role in regulating the NLRP3 inflammasome through transcription of its components, particularly by upregulating the expression of NLRP3 and pro-IL-1β, essentially acting as “priming” signals for NLRP3 activation in response to inflammatory stimuli.

Some studies suggest that prolonged NLRP3 inflammasome activation in response to crocidolite asbestos exposure can contribute to early and chronic inflammation (114). Others postulate that HMGB1, that both stimulates and is a consequence of inflammasome activation, contributes to MM development in rodents (67). However, NLRP3 deficient mice show a similar incidence of MMs when compared to wild-type mice, suggesting that NLRP3 activation may not be critical to the development of MMs (118).

#### 2.3.5 Activator transcription factor 3 (ATF3)

Activator Transcription Factor 3 (ATF3) is a stress-induced transcription factor that acts as a hub of adaptive responses in cells (119). It has been shown using human mesothelial cells that crocidolite asbestos induced increased expression of *ATF3*, indicating its participation in cell defense from fibers and particles (120). Moreover, in cells exposed to asbestos, silencing of *ATF3* increased production of inflammatory cytokines and growth factors such as IL-1B, PDGFBB, VEGF and IL-13.

### 2.4 Epigenetic control mechanisms

Asbestos fibers have been historically regarded as epigenetic as they do not act directly with DNA to form adducts or metabolites (121, 122). The most frequently studied epigenetic marker in cells is DNA methylation, a process catalyzed by DNA methyl transferases that results in covalent attachment of a methyl group to cytosine. Methylation also occurs at sites of CpG dinucleotides within the promoter regions of genes. Whereas, methylation causes condensation of chromatin, making it inaccessible for transcription, histone acetylation (addition of –COCH3) causes increased accessibility of DNA for transcription. The dynamic reversible processes of acetylation, deacetylation and methylation/demethylation control gene expression in normal and tumor cells (123).

DNA methylation profiling of MET5A mesothelial cells exposed to crocidolite or chrysotile revealed methylation at CpG sites located in genes related to migration and cell adhesion (124). Global and gene-specific DNA methylation effects of crocidolite, amosite and chrysotile fibers have been studied in immortalized human bronchial epithelial cells over a range of asbestos concentrations (125). Global DNA methylation was observed after exposures to crocidolite or amosite, but not chrysotile asbestos. Moreover, no significant changes were observed at the lowest concentrations of amphibole fibers, illustrating a threshold effect. Hierarchical clustering of gene-specific DNA methylation patterns also showed different patterns in chrysotile-exposed cells as compared to amphiboles. Examination of genome-wide methylation changes in lung cancers from smokers and individuals exposed to asbestos revealed unique changes, suggesting that methylation changes may be predictive of these risk factors (126).

Loss of function mutations of tumor suppressor genes that have been associated with cell cycle control have been reported in human and rodent MMs as discussed earlier in this perspective. For example, methylation of the *CDKN2A*/*p16INK4A* gene promoter region occurs in human MMs (127). The *CDKN2A* locus encodes the tumor suppressor proteins, p16INK4 and p14ARF, which regulate the Rb and p53 cell cycle pathways. Loss of CDK2NKB function has also been noted in lung cancers, MMs, and experimental models of mesothelioma where loss of function reflected increased numbers of tumors with decreased latency periods (128).

The methylation status and silencing of the *CDKN2A* gene has been studied in precancerous bronchial lesions from a series of 37 patients at high risk for lung cancer (129). Increases in methylation occurred with the severity of lesions, suggesting a relationship to the development of lung cancers.

The studies above illustrate the complexity of methylation changes that could be linked to gene expression governing cell defense or initiation of carcinogenic changes by asbestos fibers. The interplay between these epigenetic events and non-coding RNAs may exert protective effects or participate in oxidant-dependent signaling cascades (130).

## 3 Discussion

The likelihood and magnitude of a biologically relevant response is related to the dose of the substance to which one is exposed. In addition, other factors such as immunologic status, age of exposure, and the microenvironment, etc., influence the vulnerability and severity of exposure. A unifying concept in the biological sciences, and a fundamental tenet in toxicology, is the dose-response relationship. This principle, which has been recognized since at least the sixteenth century, holds that the likelihood and degree of a biologic response is related to the amount of the toxicant administered, and is often described by the oldest and most venerated axiom in toxicology: the dose makes the poison (131, 132). As described in *Casarett and Doull's Toxicology: The Basic Science of Poisons*, “[i]t is generally recognized that, for most types of toxic responses, a threshold exists such that at doses below the threshold, no toxicity is evident” [(133), p. 22].

Although *in vitro* or *in vivo* experimental studies do not provide precise estimates of the biologically effective dose (the actual dose that contributes to the risk of disease) that occurs under realistic exposure scenarios in humans, they do provide evidence for the existence of a threshold dose (a minimum dose that triggers minimal detectable biological effect). More specifically, *in vitro* studies using asbestos fibers over a range of concentrations demonstrated levels below which no increases in *c-jun/c-fos* gene expression and/or cell division occurred (70, 101, 134, 135).

### 3.1 Thresholds in genotoxicity

There is an obvious disconnect between toxicity, genotoxicity, and carcinogenic effects of asbestos fibers. In the majority of toxicity and genotoxicity studies, chrysotile asbestos at equal weight concentrations is more active than amphibole asbestos (23, 136). This is attributed to the positive surface charge of chrysotile rendered by Mg^2+^ interacting with negative sialic acid residues on the cell surface whereas amphiboles had a neutral or slightly negative charge (137, 138). In contrast, chrysotile asbestos is much less pathogenic in the causation of MMs than amphibole asbestos, in part because Mg^2+^ is leached from chrysotile over time resulting in its conversion to a non-reactive amorphous particle (139). In fact, its ability to cause genotoxicity, aneuploidy, and cytotoxicity due to large scale deletions in DNA (140) might explain why chrysotile asbestos is not a potent carcinogen in the development of MMs, as a dead cell cannot give rise to tumors.

Throughout the last two decades, numerous independent studies demonstrate the potential for asbestos fibers to act as genotoxic agents by inducing DNA and chromosomal damage in lung and pleural cells [reviewed in Barlow et al. (23)]. DNA damage induced by asbestos is an early event *in vitro* that may result in genetic instability, necrosis, or apoptosis at high concentrations of fibers and cell transformation at low doses (23, 141). All types of asbestos fibers are capable of mediating chromosomal and DNA damage, such as DNA breaks, cross-linking, and base lesions at high, toxic concentrations (142). Despite the overall lack of dose-response in genotoxic studies in the literature, dose-response relationships are observed in some genotoxicity studies, suggesting cell defense mechanisms such as DNA repair mechanisms and antioxidant responses. For example, non-toxic concentrations of crocidolite asbestos (1.25 and 2.5 μg/cm^2^) causes increased expression of AP-endonuclease in a dose-dependent manner in isolates of rat pleural mesothelial cells with persistent increases over a 72-h time frame (143). AP-endonuclease was induced at both non-toxic and toxic concentrations of crocidolite. Both transformed and normal cells exhibit dose-related responses to asbestos fibers as indicated by markers of DNA and chromosomal damage, including a lack of effects at lowest concentrations (21). These studies suggest NOAELs.

More recent studies of mechanisms of asbestos-induced injury and disease have focused on indirect effects that lead to DNA damage, and in some cases, the development of lung cancer or MM. These are different from the direct assault on DNA described above in that the focus is on the importance of generation of ROS. As outlined earlier, asbestos fibers were found to stimulate production of ROS though Fe-mediated and cell-mediated mechanisms *in vitro* (39, 49). Therefore, when discussing the genotoxic potential of asbestos, it is important to distinguish between primary and secondary genotoxicity. The surface properties associated with the different forms of asbestos are believed to play a major role in the primary genotoxicity of asbestos, while the excessive and persistent formation of ROS from inflammatory cells are postulated to play a role in secondary genotoxicity (23, 144). Inflammation is known to persist only at a sufficient dose, and therefore secondary genotoxicity is believed to occur at a threshold dose.

### 3.2 Thresholds in inflammation

Research has shown that exposure to long fibers of amphibole asbestos and carbon nanotubes significantly causes inflammatory responses in laboratory studies (145–148). Moreover, chronic inflammation is a critical process in the development of human MMs (149–151). Asbestos dose is a crucial determinant for triggering inflammation as high doses over short periods promote an acute neutrophil predominant inflammation whereas low doses over prolonged periods promote an alveolar macrophage predominant chronic inflammation. Of note, several studies using chrysotile and crocidolite have demonstrated levels below which no increases in gene and protein markers of inflammation and disease occur (152). The data inherently negate the legitimacy of a no threshold model as the induction of the inflammatory response contains a natural threshold for inflammatory response activation (153–155).

It is widely accepted that inflammation is a significant driver of carcinogenesis, particularly during the tumor promotion phase (156). Some of the proposed pathobiological processes for inflammation-induced carcinogenesis are through indirect DNA damage due to generation of ROS/RNS within target cells or from macrophages and other immune cells, changes in metabolism, and disruption of immune system homeostasis and function.

Inflammation is the main rate-limiting mode of action for increasing risk in inflammatory-mediated diseases, including MMs (156–160). As described above, the NLRP3 inflammasome plays a key role in initiating inflammation. The two-step process of priming and activation of the NLRP3 inflammasome are governed by several threshold mechanisms (153). Activation of the NLRP3 inflammasome requires a certain intensity and duration of stimulus to trigger its assembly and subsequent inflammatory cytokine release (161). Moreover, these triggering events, which may include sustained generation of intracellular and mitochondrial ROS, depletion of antioxidant pools, and lysosomal destabilization and rupture, also require a certain intensity and duration of stimulus. Both the priming (NFκB signaling and MAPK activation) and triggering event (the activating stimulus) must reach these threshold levels. As such, the NLRP3 inflammasome is activated by sufficiently high and prolonged exposures. This threshold system acts as a protective mechanism, preventing the body from overreacting to minor irritants encountered daily. These thresholds prevent small and brief exposures from triggering an inflammatory response while allowing sufficiently high and prolonged exposures to do so.

### 3.3 Thresholds in carcinogenicity

The existence of a threshold dose of asbestos at which no increased risk of asbestos-related disease can be observed is supported by occupational and environmental studies. Specifically, a number of published epidemiology studies have suggested that exposures to ambient asbestos concentrations of any fiber type are not associated with a significantly increased incidence of asbestos-related disease (162–170). For example, Price and Ware stated that although women's environmental exposures would likely have increased since the 1930s, with the increasing use of asbestos in the U.S., “the mesothelioma risk for women has not increased” [162, p. 111]. They noted that “[e]nvironmental exposure levels, although increasing, have not triggered a risk response in women. Therefore, those exposure levels must have been below a threshold for mesothelioma” [162, p. 111]. Similarly, Glynn et al. found that there was no increase in incidence rates of pleural mesothelioma among females in urban vs. rural areas in the U.S. between 1973 and 2012, despite measured differences of up to 10-fold or more in ambient airborne asbestos concentrations between these different geographical areas (171). According to the authors, these results suggested that ambient exposures to asbestos over a wide range of background concentrations have not significantly affected the incidence of pleural mesothelioma in the U.S. over the past 40 years.

Epidemiology studies of predominately chrysotile-exposed cohorts suggest that there is a cumulative chrysotile exposure below which there is negligible risk of asbestos-related diseases. Pierce et al. summarized NOAELs reported in the literature for predominantly chrysotile-exposed cohorts and found that the preponderance of studies showed cumulative chrysotile NOAELs for both lung cancers and MMs (172, 173). In an updated analysis incorporating epidemiologic studies published through 2022, Beckett et al. reported the lower- and upper-bound for the chrysotile NOAELs of 97–175 f/cc–yr for lung cancer and 250–379 f/cc-yr for mesothelioma (174). Conversely, epidemiological data have demonstrated a substantially elevated risk of all asbestos-related diseases, including mesothelioma, for occupations involving high cumulative exposures to amphibole asbestos or a combination of amphibole and chrysotile asbestos (175–182). Beckett et al. applied published relative potency factors for mesothelioma to the chrysotile NOAEL for mesothelioma reported to derive the best estimate NOAELs for predominately amosite- and crocidolite-exposed populations of 2–5 f/cc-yr and 0.6–1 f/cc-yr, respectively (174).

## 4 Summary and conclusions

We review here the mechanisms of asbestos-induced carcinogenicity with a focus on molecular pathways that are inhibited or modulated in cell defense from asbestos fiber exposures. These protective mechanisms are summarized within and are consistent with observations reported by others after exposures to chemical carcinogens and radiation (14–16). Simply put, humans have a cadre of defense mechanisms at the cellular and host level that maintain homeostasis and combat deleterious exposures to asbestos and other carcinogens. However, under certain conditions, such as increased vulnerability or exceeding critical response thresholds, these homeostatic mechanisms can be overwhelmed. This information and a review of the animal and human literature strongly suggest the existence of thresholds for MMs and lung cancers promoted by asbestos. Studies also illustrate the importance of asbestos type in calculation of NOAELs based upon different cellular responses to the commercial types of asbestos (chrysotile, amosite, and crocidolite) as well as their individual biodurability and size characteristics.
