# Correction: Cellular defense mechanisms against asbestos fibers

**DOI:** 10.3389/fpubh.2025.1713436

**Published:** 2025-10-29

**Authors:** Christy A. Barlow, Brooke T. Mossman

**Affiliations:** ^1^Aetia Scientific Collaborative, Boulder, CO, United States; ^2^Department of Pathology and Laboratory Medicine, Larner College of Medicine, University of Vermont, Burlington, VT, United States

**Keywords:** asbestos, elongated mineral fibers (EMPs), mesothelioma, lung cancer, threshold

The conflict of interest statement was erroneously given as “The authors declare that the research was conducted in the absence of any commercial or financial relationships that could be construed as a potential conflict of interest.” The correct conflict of interest statement is “This article was prepared and written exclusively by the authors. No outside entities provided input to the study or reviewed it prior to publication. CB and BM have served as experts in talc, silica, and/or asbestos-related litigation.”

The original version of this article has been updated.

